# Immunomodulatory effects of extracellular vesicles from mesenchymal stromal cells: Implication for therapeutic approach in autoimmune diseases

**DOI:** 10.1002/kjm2.12841

**Published:** 2024-05-07

**Authors:** Hsiu‐Jung Liao, Ping‐Ning Hsu

**Affiliations:** ^1^ Institute of Biopharmaceutical Sciences National Yang Ming Chiao Tung University Taipei Taiwan; ^2^ Department of Medical Research Far Eastern Memorial Hospital New Taipei City Taiwan; ^3^ Graduate Institute of Immunology, College of Medicine National Taiwan University Taipei Taiwan; ^4^ Department of Internal Medicine National Taiwan University Hospital Taipei Taiwan

**Keywords:** autoimmune disease, extracellular vesicle, mesenchymal stromal cell

## Abstract

Autoimmune disease is characterized by the proliferation of harmful immune cells, inducing tissue inflammation and ultimately causing organ damage. Current treatments often lack specificity, necessitating high doses, prolonged usage, and high recurrence rates. Therefore, the identification of innovative and safe therapeutic strategies is urgently required. Recent preclinical studies and clinical trials on inflammatory and autoimmune diseases have evidenced the immunosuppressive properties of mesenchymal stromal cells (MSCs). Studies have demonstrated that extracellular vesicles (EV) derived from MSCs can mitigate abnormal autoinflammation while maintaining safety within the diseased microenvironment. This study conducted a systematic review to elucidate the crucial role of MSC‐EVs in alleviating autoimmune diseases, particularly focusing on their impact on the underlying mechanisms of autoimmune conditions such as rheumatoid arthritis (RA), systemic lupus erythematosus (SLE), and inflammatory bowel disease (IBD). By specifically examining the regulatory functions of microRNAs (miRNAs) derived from MSC‐EVs, the comprehensive study aimed to enhance the understanding related to disease mechanisms and identify potential diagnostic markers and therapeutic targets for these diseases.

## 1 INTRODUCTION

Autoimmune diseases are a group of chronic inflammatory conditions wherein the body's immune system erroneously attacks its own organs and tissues, leading to inflammation and the formation of immune complexes.^1^ Accumulating evidence suggests that various factors contribute to the pathogenesis and progression of these diseases.^2^ Due to their chronic nature, patients with autoimmune diseases often require lifelong medication to mitigate disease progression. However, the extended usage of hormonal drugs or biologics not only increases the risk of adverse events but also imposes considerable economic burden. Therefore, autoimmune diseases have become a noteworthy public health concern, posing a substantial threat to the quality of life of the affected individuals.^3^

Mesenchymal stromal cells (MSCs) are multipotent stem cells capable of self‐renewal and differentiation into various cell types, including osteoblasts, chondrocytes, and adipocytes.^4^, ^5^ In addition to their remarkable differentiation potential, MSCs possess immunomodulatory capabilities for regulating both innate and adaptive immune cells. Recent findings have suggested that MSCs influence natural killer cells, dendritic cells (DCs), macrophages, B lymphocytes, and T lymphocytes by inhibiting their activation, proliferation, and differentiation into effector immune cells.^6^, ^7^, ^8^ When exposed to inflammatory stimuli, MSCs can alleviate inflammatory responses, promote tissue repair, and prevent infections by secreting a range of immune‐regulating factors.^9^ Presently, growing evidence supports that MSCs primarily exert their immunomodulatory effects through paracrine signaling, with emphasis on the release of exosomes.^10^, ^11^

Certain aspects related to the preparation of MSCs for clinical application must be considered. These include genetic instability and gene mutations that may arise during prolonged ex vivo culture. Additionally, problems such as the increased immunogenicity of differentiated cells, challenges related to therapeutic efficacy (such as low engraftment rates and the proportion of MSCs reaching target tissues), and the development of alloantibodies due to repeated administration should be addressed.^12^ This review investigates the trends related to MSC‐derived extracellular vesicles (MSC‐EVs). EVs are categorized into three subtypes based on their size and biogenesis mechanism: exosomes (50–150 nm in diameter), microvesicles (100–1000 nm in diameter), and apoptotic bodies (50–4000 nm in diameter). EVs are composed of a lipid bilayer encapsulating proteins/peptides, lipids, and genetic materials such as mRNA, microRNA (miRNA), and DNA.^13^ EVs exhibit stability under diverse physiological conditions and possess an immune‐privileged nature, making them well suited for therapeutic applications. Thus, MSC‐EVs have attracted considerable attention as promising cell‐free MSC therapy for autoimmune diseases.

MSC‐EVs exhibit markedly reduced immunogenicity but share similar immunomodulatory properties with MSCs, rendering them a representative cell‐free treatment approach. Research has revealed that EVs derived from adipose tissue‐derived MSCs (ADSC‐EVs) promote the differentiation of CD4^+^ T lymphocytes into a regulatory phenotype by encapsulating miR‐23a‐3p, which posttranscriptionally regulates TGF‐β receptor 2.^14^ Murine bone marrow‐derived MSC‐EVs (BMSC‐EVs) were demonstrated to impede T‐cell proliferation and induce cell cycle arrest through the p27kip1/Cdk2 signaling pathway.^15^ In a murine model of autoimmune uveitis, EVs derived from human umbilical cord‐derived MSCs (UC‐MSC‐EVs) exerted immunosuppressive effects by reducing T‐cell subsets and the infiltration of other inflammatory cells.^16^ Additionally, EVs derived from human embryonic stem cell derived MSCs (EMSC‐EVs) enhanced the generation of Tregs both in vitro and in vivo.^17^ BMSC‐EVs can prevent the differentiation of naive T cells into effector T cells and their subsequent activation.^18^ Moreover, BMSC‐EVs promote the apoptosis of activated T cells, inhibit the proliferation of self‐reactive lymphocytes, and increase the production of Tregs by increasing the levels of IL‐10 and TGF‐β.^19^ Bolandi et al. proposed that ADSC‐Exo loaded with miR‐10a promotes the differentiation of naive CD4^+^ T cells into Th2 cells and Tregs. Moreover, ADSC‐Exo miR‐10a can modulate Th17 and Treg differentiation by regulating Foxp3^+^ expression through the TGF‐β pathway.^20^ These findings indicate the similarity in immunosuppressive functions between MSC‐EVs and MSCs, highlighting their potential as cell‐free therapy in autoimmune diseases. Thus, the review provides a novel perspective on MSC‐EV treatment strategies for autoimmune diseases.

## 2 RHEUMATOID ARTHRITIS

Rheumatoid arthritis (RA) is a chronic systemic autoimmune disorder characterized by synovial inflammation, which leads to cartilage erosion and bone loss.^21^ RA is developed through a complex interplay between genetic and environmental factors.^22^ RA is characterized by the infiltration of inflammatory cells, such as T cells, macrophages, and B cells, and the subsequent hyperplasia of the synovial membrane and the overactivation of osteoclasts.^23^, ^24^

Current treatments for RA aim to reduce inflammation to alleviate joint damage and prevent bone loss. However, the conventional drugs such as disease‐modifying antirheumatic drugs (DMARDs), glucocorticoids, and nonsteroidal anti‐inflammatory drugs (NSAIDs) used in RA management lack specificity. Consequently, they affect various physiological pathways beyond the immune response, thereby increasing the risk of long‐term adverse events.^25^, ^26^, ^27^ Biologic DMARDs (bDMARDs) and small‐molecule compounds are alternative options, including Janus kinase (JAK) inhibitors targeting specific immune cells, cytokines, and compounds modulating cytokine pathways.^28^, ^29^ Unlike NSAIDs or glucocorticoids, which provide temporary relief from pain and inflammation, DMARDs contribute to delaying RA progression.^30^ Despite the effectiveness of DMARD therapy in the majority of patients with RA, 20%–30% either do not respond to treatment or experience considerable side effects. Moreover, biologics are not recommended for individuals with compromised immune systems or infections due to the increased risk of infection associated with their use.^31^

In individuals with RA, MSCs play a crucial role in modulating immune responses by interacting with both innate and adaptive immune cells. Currently, MSC‐based therapy is administered to patients with RA who do not respond to conventional RA treatments, and no association has been demonstrated between MSC‐based therapy and severe adverse events. Despite numerous clinical trials investigating the effects of MSC‐based therapy in patients with RA, no optimal therapeutic protocol utilizing MSCs has been established for RA.^32^ Moreover, many studies utilize allogeneic MSCs sourced from healthy donors due to the challenges involved in obtaining and expanding a sufficient quantity of autologous MSCs from patients with RA. Autologous MSCs sourced from patients with RA may have intrinsic genetic defects that could compromise their anti‐inflammatory capacities. Notably, because patients with RA require prolonged treatment to manage symptoms and prevent structural damage, the extended duration of therapy may impair the functionality of MSCs.

**FIGURE 1:**
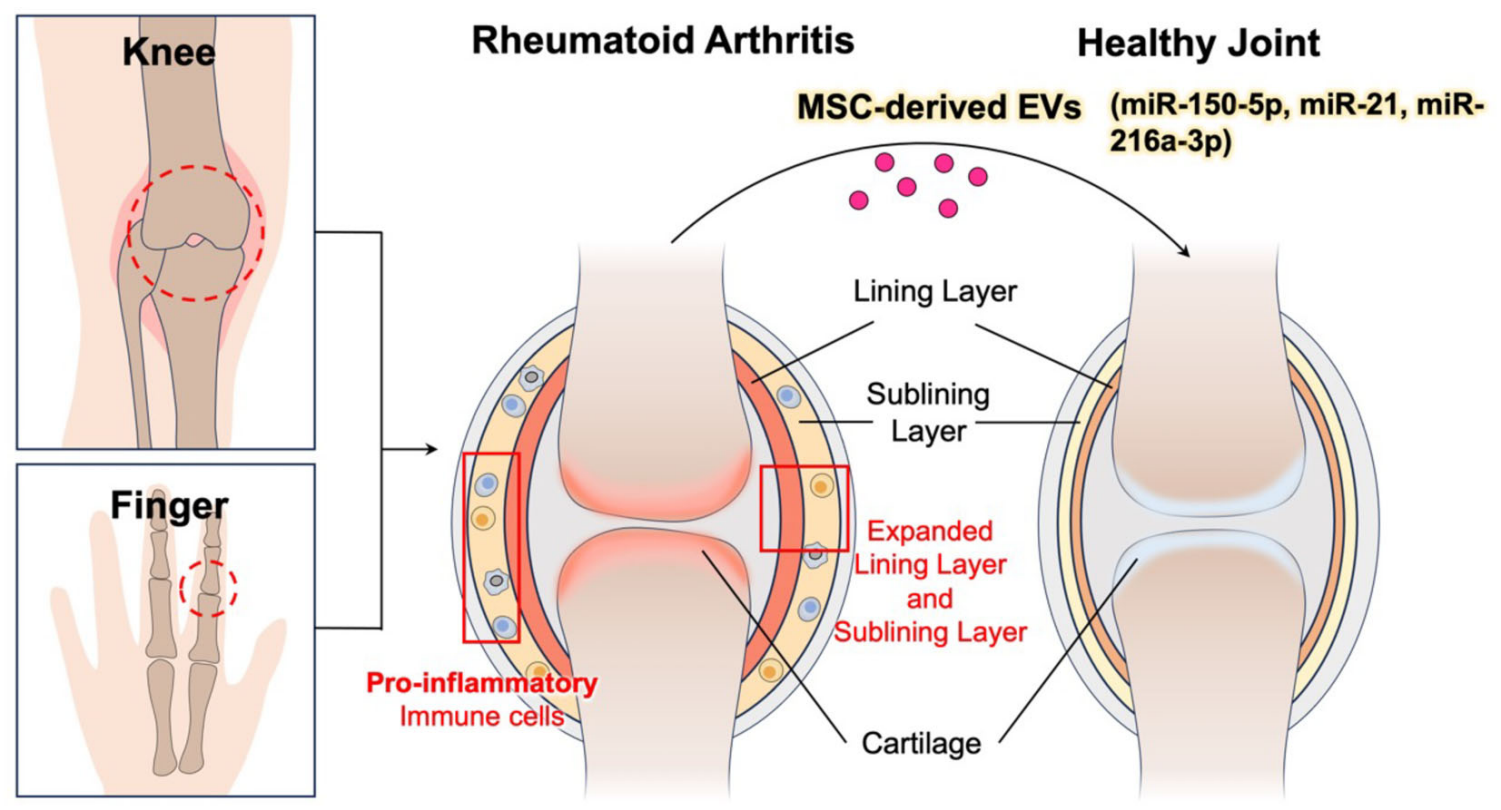
Application of MSC‐EV therapy in RA treatment. MSC‐EVs containing miR‐150‐5p, miR‐21, or miR‐216a‐3p facilitate cell‐to‐cell communication and exert immunosuppressive and immunomodulatory effects on proinflammatory immune cells and aggressive fibroblasts. These actions are pivotal in inhibiting bone destruction and promoting joint regeneration, representing key aspects of their therapeutic potential.

Studies have revealed that the mechanisms underlying the interaction between MSC‐EVs and recipient cells, immune cells, and the aggressive phenotype of synovial fibroblasts are not exclusive to specific physiological or pathological processes in RA (Figure 1). Despite this, MSC‐EVs have not been applied for RA treatment. Nevertheless, the efficacy of MSC‐EV‐based therapy has been demonstrated in experimental animal models of RA, particularly in the collagen‐induced arthritis (CIA) mouse model, which is widely employed to assess the effectiveness of MSC‐EV‐based interventions. Treatment with MSC‐EVs significantly attenuated the induction and progression of arthritis in the experimental animals. Furthermore, exosomes (exos) derived from human gingival MSCs (GMSCs) exhibited comparable or enhanced efficacy compared with GMSCs in suppressing IL‐17A and enhancing IL‐10 production, thereby reducing the incidence of arthritis and bone erosion. This effect is achieved by inhibiting the IL‐17RA‐Act1‐TRAF6‐NF‐κB signaling pathway.^33^

BMSC‐miR‐150‐5p‐Exos (Exos‐150) were found to reduce the migration and invasion of RA fibroblast‐like synoviocytes (RA‐FLS). When administered in vivo, Exos‐150 led to a reduction in hind paw thickness and lower clinical arthritic scores in mice with CIA through the inhibition of synoviocyte hyperplasia and angiogenesis.^34^ Li et al. reported that BMSC‐derived microRNA‐150‐5p‐expressing EVs alleviated CIA the delivery of miR‐21. Moreover, exosomal miR‐21 alleviated CIA by targeting the TET1/KLF4 regulatory axis.^35^ MSC‐EVs transduced with miR‐146a increased the expression of Foxp3, a transcription factor expressed in Treg cells, as well as that of TGF‐β and IL‐10 in CIA mice.^36^ MSC‐EV circFBXW7 suppressed the proliferation, migration, and inflammatory responses of RA‐FLS, thereby alleviating RA in rats through miR‐216a‐3p sponging and HDAC4 activation.^37^ Taken together, these findings suggest that MSC‐EV‐based therapy is a promising, novel, and favorable treatment modality for RA in humans.

## 3 SYSTEMIC LUPUS ERYTHEMATOSUS

Systemic lupus erythematosus (SLE) has a characteristic chronic, relapsing, and remitting nature, presenting a spectrum of symptoms ranging from mild to life‐threatening.^38^ The pathogenesis of SLE encompasses a variety of mechanisms, including the hyperfunction of T^39^ and B cells,^40^ excessive activation of the complement system,^41^ increased production of inflammatory cytokines,^42^ and impaired function of macrophages.^43^ Despite ongoing research efforts, the exact etiology of SLE remains incompletely understood. Clinically, the standard treatment for SLE includes glucocorticoids, antimalarials, and immunosuppressive drugs. Although a combination of these medications has led to improvements in patient outcomes, they exhibit drawbacks such as imprecise targeting, the necessity of high doses, prolonged administration durations, and high recurrence rates.^44^, ^45^, ^46^ Studies have indicated that infection (33.2%), renal degeneration (18.7%), encephalopathy (13.8%), and cardiovascular disease (11.5%) are the primary causes of mortality in patients with SLE. However, with the widespread use of hormonal drugs, infection has become the leading cause of death in individuals with SLE.^47^ In recent years, the paracrine action of MSCs has received considerable attention. This action occurs through the interaction of various soluble immune regulatory factors, growth factors, noncoding RNA, and proteins with MSC‐EVs.^48^, ^49^

**FIGURE 2:**
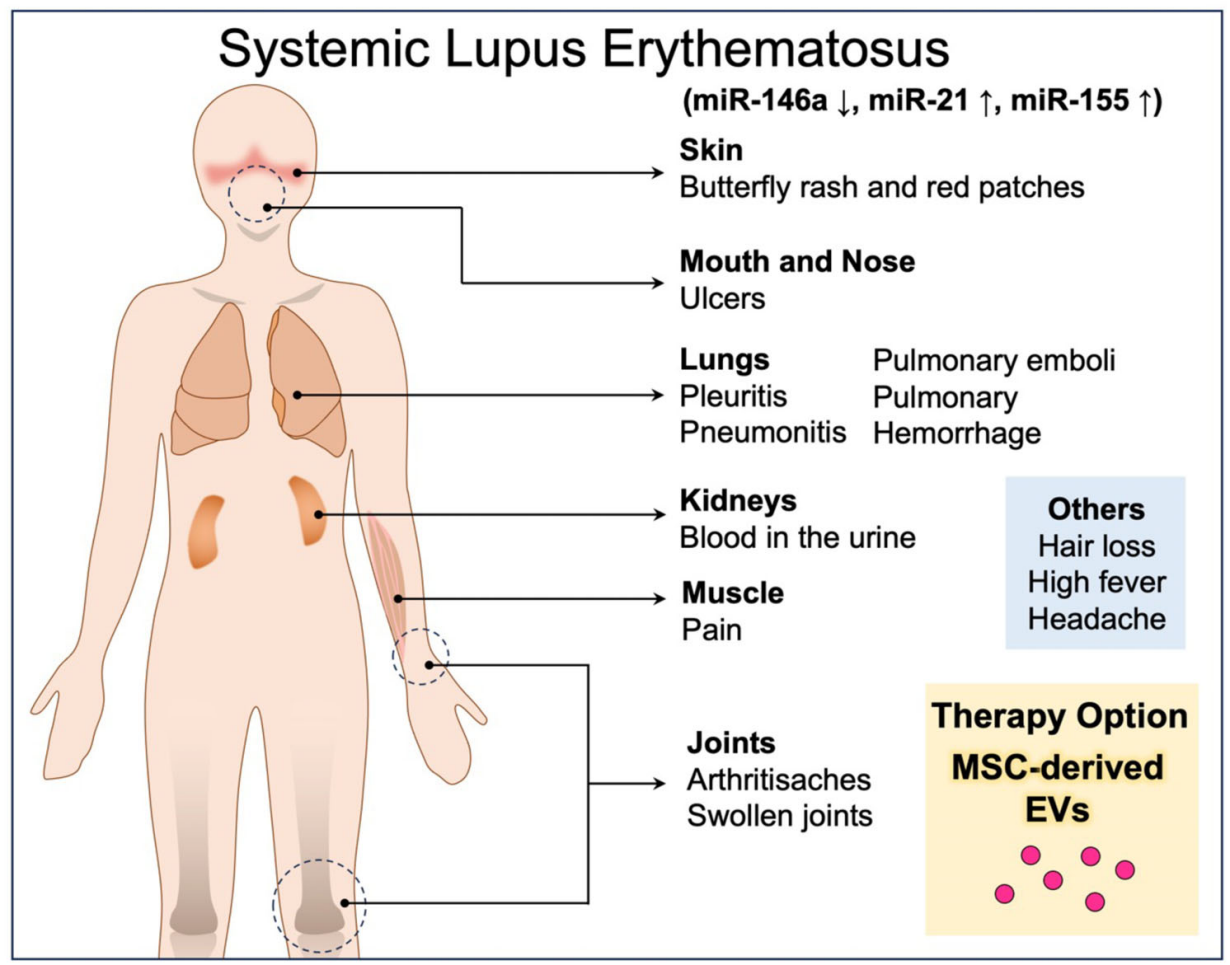
Role of MSC‐EVs in immune regulation and regeneration in SLE. Levels of miR‐146a, miR‐21, and miR‐155 in serum are significantly altered in patients with SLE. These EVs mimic the effects of MSCs and exert potent functions by modulating immune pathways, enhancing effector cell migration and proliferation of effector cells and reducing apoptosis. However, evidence regarding the administration of MSC‐EVs in SLE animal models and clinical trials is limited. Despite EVs eliciting a reduced immune response and possessing a higher safety profile compared with MSC cell therapy in patients with SLE, obstacles to their clinical implementation remain.

MiRNAs, endogenous single‐stranded noncoding RNAs, play a crucial role in both innate and adaptive immune responses by modulating cell proliferation, differentiation, and apoptosis.^50^ Disruption and dysfunction of miRNAs are associated with immune response interference, leading to the release of inflammatory cytokines, initiation of autoantibody production, and exacerbation of SLE.^51^, ^52^ In patients with SLE, miR‐146a expression in serum exosomes is significantly reduced, suggesting miR‐146a expression as a biomarker. Furthermore, exosomal miR‐21 and miR‐155 expression is upregulated, whereas miR‐146a expression is downregulated in individuals with SLE compared with healthy controls. Moreover, miR‐21 and miR‐155 levels are elevated in patients with SLE having lupus nephritis (LN) compared with those without LN (non‐LN), with a positive correlation observed with proteinuria. The expression levels of miR‐21 demonstrated a negative association with anti‐SSA/Ro antibodies, whereas miR‐146a expression was inversely associated with anti‐dsDNA antibodies. These findings suggest that the expression levels of exosomal miR‐21 and miR‐155 serve as biomarkers for the diagnosis of SLE and LN.^53^ Additionally, the reduction in miR‐146a expression can induce MSC senescence by targeting TRAF6 and inhibiting the activation of the NF‐κB signaling pathway.^54^ Furthermore, single‐nucleotide polymorphisms (SNPs) in miRNA‐146a may be correlated with immune‐system dysfunction. This may be attributed to the correlation among SNPs in miRNA‐146a and its target gene, interleukin‐1 receptor‐associated kinase 1 (IRAK1), and disease susceptibility, clinical manifestations, and disease progression in patients with SLE. Exosomal miR‐146a plays a pivotal role in regulating innate immunity through IRAK1,^55^ indicating its potential as a key biomarker of SLE. Furthermore, as a therapeutic approach, loading miRNA biomarkers into MSC‐EVs may enhance the anti‐inflammatory microenvironment. Taken together, the results indicate that MSC‐EVs exert robust anti‐inflammatory and immunomodulatory effects, making them an ideal therapeutic strategy for SLE, particularly in patients with refractory and severe SLE resistant to hormonal and immunosuppressant drugs. Therefore, the combination of MSC‐EVs and immunosuppressants may be a more effective therapeutic strategy for managing SLE, offering patients a safer and more effective alternative to traditional treatments (Figure 2).

## 4 INFLAMMATORY BOWEL DISEASE

Inflammatory bowel disease (IBD), comprising ulcerative colitis (UC), and Crohn's disease (CD), develops from a complex interplay of genetic susceptibility, environmental factors, intestinal microbiota, and the immune system, with immune factors playing a crucial role. The past few decades has witnessed a global surge in IBD incidence.^56^ Patients with IBD may experience extracolonic manifestations such as primary sclerosis, cholangitis, and arthritis, and they have increased risks of complications such as colon cancer, coronary artery disease, osteoporosis, and venous thrombosis compared with the general population.^57^ Traditional clinical treatments for IBD are divided into three categories: traditional therapeutic drugs (e.g., 5‐aminosalicylic acid, glucocorticoids, and immunosuppressants), biological agents (e.g., antitumor necrosis factor‐α drugs, insulin receptor antagonists, and cytokine inhibitors), and new small‐molecule drugs (e.g., selective Janus kinase inhibitors and sphingosine‐1‐phosphate receptor modulators). Despite their effectiveness, these treatments often have complex maintenance requirements and are associated with various adverse reactions, including increased mortality risk.^58^ The use of immunosuppressants increases the risk of opportunistic infections, and adverse effects such as intolerance or potential bone marrow/liver toxicity may lead to treatment cessation in a quarter of patients.^59^ Although ileocolectomy serves as a common surgical strategy for CD, it rarely provides a cure, as new lesions typically develop rapidly at the anastomosis, with documented risks of urinary incontinence.^60^ Given these challenges, the development of new treatments that can effectively ameliorate IBD conditions without the risk of incontinence is urgently required.

MSC therapy is promising for IBD treatment. However, addressing the limitations associated with the inherent function of naive seeding cells and achieving effective targeting within the intestines remain continuous endeavors. Moreover, the conventional intravenous injection method is systemic, with drawbacks such as inadequate targeting and rapid metabolism. By contrast, as an innovative medical approach, MSC‐EVs potentially possess remarkable immunomodulatory properties without the common side effects associated with the current antitumor necrosis factor drugs and stem cell therapies.

In a phase I clinical trial involving umbilical cord‐derived EV administration, Hojjatollah Nazari observed positive responses in four patients 6 months after therapy initiation. Among those who received exosome injections, three patients achieved complete healing, whereas one reported no improvement with continued discharge from the fistula site. Furthermore, all five patients (100%) reported neither systemic nor local adverse effects.^61^ Furthermore, in vivo animal studies have demonstrated comparable efficacy between ADSCs and EVs for suppressing inflammation in the DSS colitis model. Their suppressive effects were achieved by enhancing the colon length, reducing colonic bleeding, and alleviating the histologic severity of inflammation through the inhibition of the JAK, JNK 1/2, and STAT3 signaling pathways and reactive oxygen species activity.^62^, ^63^ Moreover, in the IBD microenvironment, an increase in the recruitment of innate immune cells, including DCs, neutrophils, monocytes, macrophages, and T cells, was observed after therapy initiation. Thus, MSC‐EVs exhibit a strong association with macrophages in IBD. The enhancement of IL‐10 production from macrophages partly contributes to the positive effect of MSC‐Exos on regulating various biological processes associated with the anticolitic properties of MSC‐Exos. Specifically, metallothionein‐2 in MSC‐Exos plays a crucial role in suppressing inflammatory reactions.^64^ Moreover, in experimental colitis mice, the application of olfactory ecto‐mesenchymal stem cells (OE‐MSCs) effectively reduced the severity of the disease. Subsequent treatment with OE‐MSC‐Exos led to a significant reduction in Th1/Th17 subpopulations coupled with a notable increase in Treg cells.^65^

EVs derived from UC‐MSCs are promising for alleviating colitis in mice. In the IBD group, the gene expression levels of TNF‐α, IL‐1β, IL‐6, NAe1, E2M, and Uba3 were significantly upregulated, whereas the expression levels of IL‐10 and IP‐10 were downregulated. Conversely, the gene expression profile in the group treated with hUC‐MSC‐Exos contrasted with that in the IBD group.^66^ In a recent study, human ADSC‐Exos were demonstrated to stimulate the proliferation and regeneration of Lgr5‐positive intestinal stem cells and epithelial cells. Additionally, hADSC‐Exos ameliorated TNF‐α‐induced inflammatory damage in mouse colon organoids. These findings suggest that hADSC‐Exos is a potential treatment option for IBD because they contribute to maintaining intestinal integrity and activating intestinal epithelial cells.^67^

**FIGURE 3:**
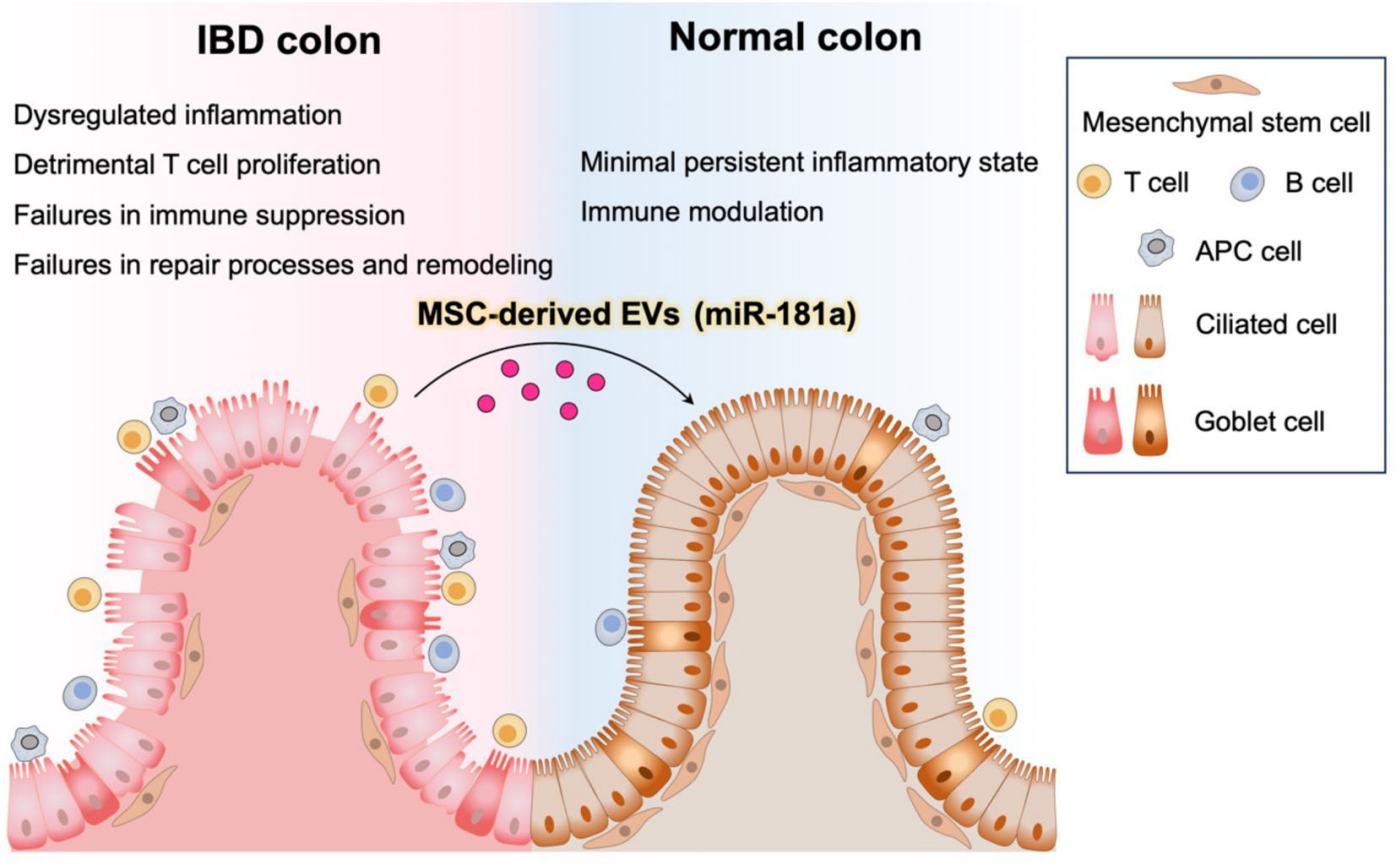
Interaction mediated by MSC‐EVs in the intestinal microenvironment. In the intestinal microenvironment, MSC‐EVs play a crucial role in preserving intestinal homeostasis, fostering immune cell maturation, and modulation. Conditions such as IBD disrupt the equilibrium of the host‐commensal system, leading to impaired immune responses and compromised intestinal barrier integrity. MSC‐EVs harboring miR‐181a engage in direct or indirect interactions with immune cells, intestinal epithelial cells, and gut microbiota, thereby modulating anti‐inflammatory responses, enhancing mucosal barrier integrity, and restoring the microbiota composition.

The administration of EVs derived from unprimed MSCs may not effectively improve the clinical and histomorphometric indices of colitis. Nevertheless, the enhancement of the disease activity index and histomorphometric score was observed. Conversely, the administration of EVs derived from stimulator‐conditioned MSCs significantly improved all clinicopathological parameters associated with IBD. Nie et al. proposed an immunosuppressive strategy for treating IBD that utilized adhesive microparticles loaded with MSC‐EVs carrying the IL‐27 gene. When administered rectally, MSC‐IL‐27 EVs significantly inhibited inflammation and improved the integrity of the intestinal epithelial barrier, thereby mitigating IBD‐associated damage.^68^ Moreover, in vivo experiments using a DSS‐induced colitis model revealed that the intragastric administration of exosomal miR‐181a derived from BMSCs upregulated the expression of colonic tight junction proteins and alleviated symptoms associated with DSS‐induced colitis in mice. Furthermore, alterations in the composition and structure of the gut microbiota were observed.^69^ Addressing these challenges involves integrating novel MSC‐EV approaches and technologies as well as making informed decisions about the optimal MSC source to counteract the multifactorial pathophysiological mechanisms implicated in IBD. Recent studies have demonstrated that MSC‐EVs primarily alleviate gut inflammation by repairing the intestinal mucosal barrier and maintaining the immune balance, suggesting that MSC‐EV is a promising treatment for IBD (Figure 3). Additionally, establishing effective mass production and standardization protocols is crucial for promoting the clinical application of these new products. Cutting‐edge technological advancements, such as nanotechnology‐based hydrogels, play a vital role in optimizing the application of these therapeutic agents.

## 5 CONCLUSIONS

In recent years, MSC‐EVs have been extensively explored for their application as therapeutic interventions in autoimmune diseases. These EVs offer promising therapeutic mechanisms, including immunomodulation, direct antimicrobial effects, and tissue repair. They exert their effects through various molecules such as mRNAs, proteins, and miRNAs. Compared with traditional MSC‐based therapies, MSC‐EVs represent a cell‐free alternative that is preferred due to their reduced risk of triggering immune responses, tumorigenicity, and overall enhanced safety profile.

Currently, 15 clinical trials involving MSC‐EVs have been registered on ClinicalTrial.gov, although they are yet to commence.^70^ Despite the increased safety of EV‐based therapies, specific concerns must be addressed for their clinical application. Notably, considerable variation exists in the related research, highlighting the need for standardized procedures for MSC expansion and EV purification methods. Establishing protocols and conducting potency tests are critical for ensuring the clinical‐grade quality of exosomes or EV preparations. However, further research is necessary to comprehensively understand the immunomodulation mechanisms and identify the EV cargo responsible for cellular‐level alterations specific to clinical conditions. Additionally, comprehensive toxicity profiling, including assessments of immunological responses, is essential for all EV preparations. In summary, MSC‐EVs are a promising alternative to MSC‐based therapy and offer a new biological approach for the clinical treatment of autoimmune diseases. Table 1 presents a summary of animal and clinical studies on RA, SLE, and IBD. Nonetheless, certain challenges persist and must be addressed through foundational research, involving elucidating the underlying mechanisms and enhancing the therapeutic effectiveness of MSC‐EVs.

**TABLE 1:** Summary of clinical and animal studies on MSC‐EVs therapy.

Cell source	Model	Results	Reference
Rheumatoid arthritis (RA)			
Human GMSCs	CIA animal model	GMSC‐Exo demonstrated comparable or higher efficacy compared with GMSCs in suppressing the expression of IL‐17A and increasing the expression of IL‐10, thereby reducing the incidence of arthritis and bone erosion.	33
Mouse BMSCs	CIA animal model	BMSC‐miR‐150‐5p‐Exos (Exo‐150) reduced the migration and invasion of RA fibroblast‐like synoviocytes. Exo‐150 reduced hind paw thickness and clinical arthritic scores by inhibiting synoviocyte hyperplasia and angiogenesis.	34
BMSCs	CIA animal model	EVs derived from BMSCs alleviated CIA by delivering miR‐21. Exosomal miR‐21 alleviated CIA by targeting the TET1/KLF4 regulatory axis.	35
MSCs	CIA animal model	MSC‐Exos transduced with miR‐146a increased the expression of FOXP3, TGFβ, and IL‐10 in CIA mice. miR‐155 overexpression further increased the expression of RORγt, IL‐17, and IL‐6.	36
BMSCs	CIA animal model	Exosomal circFBXW7 derived from BMSCs inhibited the proliferation, migration, and inflammatory response of RA‐FLSs and alleviated damage in RA rats. This effect was achieved through the sequestration of miR‐216a‐3p and subsequent activation of HDAC4.	37
Systemic lupus erythematosus (SLE)			
Serum	Patients with SLE (biomarker)	Exosomal miR‐21 and miR‐155 were upregulated, whereas miR‐146a was downregulated in patients with SLE compared with healthy controls.	53
BMSCs	Patients with SLE	Reduced miR‐146a levels may induce MSC senescence by targeting TRAF6 and inhibiting NF‐κB signaling pathway in patients with SLE.	54
Serum	Patients with SLE	Variations in miRNA‐146a, specifically SNPs, may be associated with the onset of immune‐system dysfunction. This association is attributed to the relationship between SNPs in miRNA‐146a and its target gene, IRAK1, and its impact on the susceptibility, clinical manifestations, and progression of disease in the patients.	55
Inflammatory bowel disease (IBD)			
Human UC‐MSCs	Patients with IBD, clinical trial phase I	Within the first 6 months of therapy initiation, four patients responded positively. Among those who received exosome injections, three reported complete healing, and one reported no improvement with ongoing discharge from the fistula site. All five patients (100%) reported no systemic or local adverse effects.	61
Human ADSCs	DSS‐induced colitis animal model	ADSCs and ADSC‐EVs reduced colonic lymphocyte infiltration and disease severity by inhibiting JAK, JNK 1/2, and STAT3 signaling pathways.	62
Human P‐MSCs	TNBS‐induced colitis animal model	P‐MSC‐EV alleviated TNBS‐induced colitis by suppressing inflammation and oxidative stress.	63
Human BMSCs	DSS‐induced colitis animal model	The increased production of IL‐10 by macrophages partly contributed to the positive impact of MSC‐Exos on the regulation of various biological processes associated with the anticolitic properties of MSC‐Exos. Specifically, metallothionein‐2 in MSC‐Exos played a crucial role in suppressing inflammatory reactions.	64
OE‐MSCs	DSS‐induced colitis animal model	OE‐MSCs significantly mitigated disease severity. Following treatment with OE‐MSC‐Exos, a notable decrease in Th1/Th17 subpopulations was observed, whereas Treg cells exhibited a remarkable increase.	65
Human UC‐MSCs	DSS‐induced colitis animal model	Human UC‐MSC‐Exos significantly alleviated IBD symptoms in mice by increasing the gene expression levels of TNF‐α, IL‐1β, IL‐6, NAe1, E2M, and Uba3.	66
Human ADSCs	TNF‐α induced inflammatory damaged mouse colon organoids	hADSC‐Exos enhanced the proliferation and regeneration of Lgr5^+^ intestinal stem cells and epithelial cells and alleviated TNF‐α‐induced inflammatory damage in mouse colon organoids.	67
MSC‐IL‐27 EVs	DSS‐induced colitis animal model	When administered rectally, MSC‐IL‐27 EVs significantly inhibited inflammation and enhanced the integrity of the intestinal epithelial barrier, thereby alleviating damage associated with IBD.	68
Human BMSCs	DSS‐induced colitis animal model	Exosomal miR‐181a derived from BMSCs relieved experimental colitis by enhancing intestinal barrier function through its anti‐inflammatory effects and modulated the gut microbiota composition.	69

Abbreviations: ADSCs, adipose‐derived MSCs; BMSCs, bone marrow derived MSCs; CIA, collagen‐induced arthritis; DSS, dextran sulfate sodium; GMSCs, gingival MSCs; OE‐MSCs, olfactory ecto‐MSCs; P‐MSCs, placental MSCs; TNBS, trinitrobenzene sulfonic acid; UC‐MSCs, umbilical cord‐derived MSCs.

## CONFLICT OF INTEREST STATEMENT

The authors declare no conflict of interest.
